# Pharmacokinetic and pharmacodynamic evaluation study of etomidate: a randomized, open-label, 2-period crossover study in healthy Chinese subjects

**DOI:** 10.1038/s41598-024-57581-2

**Published:** 2024-03-25

**Authors:** Ying Ding, Nan-nan Chu, Rui Wang, Wei Qin, Yun-fei Shi, Zhen-zhong Qian, Bo Liu, Qing He

**Affiliations:** 1 Drug Clinical Trial Institution, Wuxi People’s Hospital Affiliated with Nanjing Medical University, Wuxi, 214023 Jiangsu China; 2 Jiangsu Nhwa Pharmaceutical CoLtd, Xuzhou, Jiangsu China

**Keywords:** Drug discovery, Medical research

## Abstract

Etomidate is a sedative and hypnotic drug through intravenous administration that act on the central nervous system through GABA (Gamma-Amino Butyric Acid) receptors, which is widely used in anesthesia induction and maintenance and long-term sedation in severe patients. The study aimed to evaluate the pharmacokinetic and pharmacodynamic properties of two etomidate fat emulsions after administration through the intravenous infusion pump in healthy Chinese subjects. A randomized, open-label, 2-period crossover study was performed in 52 healthy subjects. The wash-out period was 7 days. Blood samples and pharmacodynamic index values were collected at the specified time points. Etomidate concentrations were measured using validated liquid chromatography-tandem mass spectrometry. Pharmacokinetic parameters were analyzed using a non-compartment model method. Pharmacodynamic parameters were calculated using pharmacodynamic index values. The study also evaluated the safety of the etomidate. Both the pharmacokinetic parameters and pharmacodynamic parameters result of the test and reference formulation were very similar. The 90% confidence intervals (CI) of the geometric least-squares mean (GLSM) ratios of the test to reference formulation were 91.33–104.96% for the maximum plasma concentration (C_max_), 97.21–102.03% for the area under the plasma concentration time curve from time 0 to the time of the last measurable concentration (AUC_0–t_), and 97.22–102.33% for the area under the plasma concentration time curve from time 0 to infinity (AUC_0–∞_). Meanwhile, the 90% CI of the GLSM ratios of the test to reference formulation were 102.28–110.69% for the minimal BIS value (BIS_min_), 99.23–101.17% for the area under the BIS time curve from time 0–60 min after administration (BISAUC_0–60 min_), respectively. The 90% CI of these pharmacokinetic and pharmacodynamic parameters all fall in the accepted bioequivalence range of 80.00–125.00%. No serious adverse events occurred during the study. This study has shown that the etomidate fat emulsion test and reference formulation had similar pharmacokinetic and pharmacodynamic characteristics in vivo. The two formulations exhibited good safety and well-tolerance.

**Clinical trials registration number:** http://www.chinadrugtrials.org.cn/index.html. # CTR20191836.

## Introduction

Intravenous anesthetics have systemic sedative, hypnotic, and analgesic effects on the central nervous system through blood circulation. Compared with inhaled anesthetics, intravenous anesthetics have the outstanding advantages of no respiratory tract stimulation, rapid recovery, high recovery quality, unlimited application, simple equipment, and no environmental pollution. Therefore, it has been playing an important role in clinical anesthesia.

Etomidate, a non-barbiturate, is a sedative and hypnotic drug through intravenous administration that acts on the central nervous system through GABA (Gamma-Amino Butyric Acid) receptors. Etomidate has been widely used in clinic since it was invented in 1972^1^. Etomidate can slightly expand coronary artery, reduce intracranial pressure and maintain cerebral perfusion. Meanwhile, etomidate also has brain protective effect and the pharmacokinetic characteristics of etomidate make patients wake up quickly after single injection or continuous infusion. Therefore, etomidate is widely used in anesthesia induction and maintenance, and long-term sedation in severe patients. Etomidate is mainly divided into water and fat emulsion, the main difference between the two formulations is the different osmotic concentration. Compared with water formulation, fat emulsion is closer to the range of physiological osmotic concentration, so the probability of injection pain and phlebitis of fat emulsion is lower, which is also the most commonly used dosage form in clinic.

During injection, etomidate of fat emulsion quickly separates from the oil particles. This can be reflected from the fact that the etomidate plasma concentration of fat emulsion is the same as its aqueous form^2^. The plasma protein binding rate of etomidate is about 75%, mainly binding to albumin. The protein binding rate decreases in patients with renal insufficiency or chronic liver injury^3^. Due to the high solubility of etomidate in fat, its central and peripheral distribution volumes are relatively large, at 4.5 L/kg and 74.9 L/kg, respectively^4^. Some studies showed that the pharmacokinetics of etomidate conformed to the open three compartment model. It is generally believed that the rapid, intermediate, and slow decrease of plasma etomidate corresponds to three stages, namely distribution to hyperperfusion tissues, redistribution to peripheral tissues (mainly muscle), and terminal metabolism. With the redistribution of etomidate to the peripheral compartments and the beginning to dominate the plasma concentration, the hypnotic effect of intravenous injection of 3 mg/kg etomidate gradually ended. The metabolism of etomidate mainly depends on hepatic esterase activity, and etomidate is hydrolyzed into a carboxylic acid and ethanol leaving group. Carboxylate metabolites are primarily excreted through urine, with minimal amounts in bile. The metabolism of etomidate is eliminated quickly in the human body, with a total plasma clearance rate of 15–20 mL/kg/min and a terminal metabolic half-life of 2–5 h. Due to decreased protein binding and clearance rate, elderly or diseased patients typically require lower doses of etomidate^5^. These pharmacokinetic characters for etomidate show that it is suitable for continuous infusion and has a shorter half-time than propofol^6^.

To reduce drug costs, the alternative clinical use between generic and original drugs has recently been a focus of attention. Although etomidate injectable emulsion (Lipuro®, B.Braun Melsungen AG, Germany) was approved for listing in China in 2007, due to the difficulties in drug development, there is no domestic generic etomidate in the Chinese market at present. Meanwhile, although the pharmacokinetics of etomidate has been reported, there is a lack of papers on the pharmacokinetic and pharmacodynamic study of etomidate in healthy populations. This was the first reported bioequivalent study of etomidate which aimed to assess the pharmacokinetic and pharmacodynamic characteristics of etomidate after intravenous administration of the test formulation or the reference formulation and to evaluate safety and tolerability in healthy Chinese subjects. This study results will provide support for the marketing of generic formulation of etomidate in China, and may also shed light on the clinical study design and conduction of etomidate bioequivalence.

## Subjects and methods

### Subjects

Male or female participants in this study should be between 18 and 55, with a body mass index (BMI) between 19.0 and 26.0 kg/m^2^. Each subject must go through a detailed disease history inquiry, comprehensive physical examination, vital sign measurement, clinical laboratory examination (including blood routine, urine routine, blood biochemistry, blood coagulation, and virology examination), chest X-ray, abdominal B ultrasound and 12 lead ECG examination. All these examination results must be normal or evaluated by the research doctor as abnormal without clinical significance before the subject can be included in this study. The special exclusion criteria was that subjects were excluded if they had previous dyspnea or suspected airway difficulties (e.g. modified Mallampti grade III–IV, congenital macroglossia, mandibular dysplasia), or severe apnea syndrome. Other important exclusion criteria were followed as : subject had a history of alcohol abuse; the subject's urine medication and nicotine screening were positive; subject had a history of allergy to soybeans, peanuts, eggs or egg products; subject had a history of orthostatic hypotension; subject had previously donated blood or experienced acute blood loss (more than 400 mL) within the three months before the screening; subject has taken any medicine, Chinese herbal medicine, or vitamin products within 2 weeks prior to screening; subject has participated in other clinical trials within three months prior to screening. The female subjects did not breastfeed, and their pregnancy test results were negative. During the study period, all participants were not allowed to have fertility planning and were willing to take appropriate contraceptive measures. Clinical researchers provided detailed information on the research purpose, process, and risks to all participants, and each participant voluntarily signed a written informed consent form before participating in the study.

### Study design

The study was conducted in compliance with Good Clinical Practice guidelines and the ethical principles of the Declaration of Helsinki. The study protocol and informed consent form were approved by the independent ethics committee of Wuxi People’s Hospital Affiliated with Nanjing Medical University (Wuxi, Jiangsu, China).

This study was an open-label, randomized, 2-period crossover, single-dose pharmacokinetic and pharmacodynamic study in healthy adult male and female subjects. 52 healthy adult subjects were included and randomly assigned to two treatment sequences in a 1:1 ratio. Because etomidate is one particular type of study drug, the administration of etomidate was carried out in the operating room. Due to the limited number of operating beds, 52 healthy adult subjects were divided into 5 groups and every group received the study drug in different days. After overnight fasting for at least 10 h, all subjects from different batches received a single dose of etomidate medium/long-chain fat emulsion injection (Test formulation, manufactured by Jiangsu Enhua Pharmaceutical Co. Ltd, China) or etomidate injectable emulsion (Reference formulation, Lipuro, manufactured by B.Braun Melsungen AG, Germany) through the intravenous infusion pump. Although the drug names of the test and reference formulations were different, the formulas of the two formulations were slightly different, but both used medium/long-chain fat emulsions as carriers. The specifications of test formulation and reference formulation were 10 mL:20 mg. The rate of infusion pump was 5 μg/kg/min and continuous administration time was 30 min ± 0.5 min. In the morning before administration, every subject was weighed under fasting conditionto calculate the dosage. Subjects were prohibited from drinking water for 2 h before and after administration. Subjects received standard lunch and dinner at 4 and 10 h after administration. The trial procedures for the two treatments were the same and the washout period was 7 days. Throughout the entire study period, safety assessments were conducted on all subjects.

### Pharmacokinetic sample collection and analysis methods

Vacuum tubes using sodium fluoride/heparin as anticoagulant were used to collect blood samples for the determination of etomidate plasma concentrations. The blood collection points were within 1 h before administration and at 10, 20, 25, 30, 35, 45 min, and 1.0, 1.5, 2.0, 4.0, 6.0, 8.0, 12.0, 16.0, 24.0 h after administration. Blood sample centrifugation needed to complete within 1 h of blood collection, with centrifugation conditions of 1700*g* at 2–8 °C for 10 min. Then the plasma sample needed to be separated into two tubes, one for measurement and the other for backup. The plasma samples were stored in a − 80 °C refrigerator at the clinical study center until transported to the analysis laboratory (Beijing Scinovo Laboratories Co. Ltd., Beijing, China) for etomidate concentration analysis.

Plasma samples were determined at analysis laboratory and analyzed by HPLC–MS/MS methods. The detection method was fully validated by multiple evaluations of its specificity, sensitivity, accuracy, within-batch precision, between-batch precision, and stability. The standard curve range for etomidate was 0.1–200 ng/mL.

### Pharmacodynamic indicator evalution

The pharmacodynamic evaluation of etomidate is to evaluate its anesthetic / sedative effect. At present, scoring method or instrument method is often used to evaluate anesthetic/sedative effect, such as Modified Observer's Assessment of Alertness/Sedation (MOAA/S) score, Ramsay score, Bispectral Index (BIS), EEG entropy (EEM) and other methods. Among these methods, the role of BIS in monitoring the depth of anesthesia has been widely recognized. Therefore, BIS was used as pharmacodynamics index in the study. In addition, modified MOAA/S score was considered as a referable pharmacodynamics index.

Before and within 1 h after administration, each subject was equipped with BIS monitoring system. The BIS value was recorded by the subjects within 5 min before the start of administration (if it was recorded multiple times within 5 min before administration, the mean value was taken), and BIS values were recorded every 1 min within 60 min after administration. The sedation depth of the subjects was evaluated by anesthesiologists with modified MOAA/S score. The evaluation was conducted within 5 min before the beginning of administration, and every 15 min within 60 min after administration until the end of sedation.

### Pharmacokinetic and statistical analysis

We used a non-compartment model to analyze pharmacokinetic parameters in Phoenix WinNonlin software (Pharsight Corporation, California; version 8.0). The primary pharmacokinetic parameters included C_max_, the time to maximum plasma concentration (T_max_), AUC_0–t_, AUC_0–∞_, and the terminal elimination half-life (t_1/2_). C_max_ and T_max_ were actual measured values. AUC_0–t_ was calculated using the linear/log trapezoidal method. AUC_0–∞_ was calculated as AUC_0–∞_ = AUC_0–t_ + Ct/λz, where Ct was the last detectable concentration and λz was the elimination rate constant. λz was estimated by linear least-squares regression analysis for the concentration–time data obtained from the terminal log-linear phase. t_1/2_ was calculated as 0.693/λz.

After logarithmic conversion of the main pharmacokinetic parameters (C_max_, AUC_0–t_, and AUC_0–∞_) of the two formulations, ANOVA was performed. The 90% CIs of GLSM ratio of the test to reference formulation were calculated to evaluate pharmacokinetic bioequivalence. If the 90% CIs were within the range of 80.00–125.00%, the two formulations were considered to be pharmacokinetic bioequivalent. A Non-parametric test method (Paired Wilcoxon method) was used to compare the T_max_ between the two formulations_._

### Pharmacodynamic and statistical analysis

The primary pharmacodynamic parameters included the minimal BIS value (BIS_min_), the area under the BIS time curve from time 0–60 min after administration (BISAUC_0–60 min_), the time to the minimal BIS value (t-BIS_min_), and modified MOAA/S score. BIS_min_ and t-BIS_min_ were obtained from the observed values from BIS monitoring system. BISAUC_0–60 min_ was calculated by linear trapezoidal rule. Modified MOAA/S score was obtained directly through the evaluation of anesthesiologists.

The main pharmacodynamic parameters (BIS_min_ and and BISAUC_0–60 min_) between the two formulations were statistically analyzed using the ANOVA method. The 90% CIs of GLSM ratio of the test to reference formulation were calculated to evaluate pharmacodynamic parameters. If the 90% CIs were within the range of 80.00–125.00%, the pharmacodynamic parameters of the two formulations were considered to be meet the common bioequivalence interval. For t-BIS_min_ between the two formulations_,_ a non-parametric test method (Paired Wilcoxon method) was used. Further, for the Modified MOAA/S score, only descriptive summary analysis was conducted.

### Safety assessment

During the whole study, vital signs, physical examinations, 12-lead ECG, and clinical laboratory tests were conducted at predefined time points to evaluate the safety and tolerance of etomidate. Each subject was required to undergo vital signs measurements (including blood pressure, heart rate, and body temperature) within 1 h before and 2, 8, and 24 h after administration in each treatment period. During the screening period and at the end of the study, each subject was required to undergo a physical examination, 12-lead ECG, and clinical laboratory examination. In addition, throughout the trial process, relevant information on adverse events (AEs) was obtained through clinical observations by research doctors and spontaneous reports from volunteers.

### Ethics approval

Before the study was carried out, the independent ethics committee of Wuxi People’s Hospital Affiliated with Nanjing Medical University reviewed and approved by the study with the approval number of 2019LLPJ-I-23. The study was performed consistent with Good Clinical Practices and the ethical principles of Helsinki Declaration.

### Consent to participate

All subjects voluntarily signed informed consent forms before they participated in the study.

## Results

### Study population

A total of 52 healthy adult subjects (32 male and 20 female) participated in this study. The baseline demographic data and characteristics of the study population are shown in Table 1. The demographic details were as follows (mean [SD]: age was 30.1 (8.10) years (range, 20–50 years), weight 62.22 (6.858) kg (range, 50.0–76.6 kg), height 165.78 (7.239) cm (range, 155.2–184.1 cm), and body mass index 22.62 (1.777) kg/m^2^ (range, 19.6–26.0 kg/m^2^). These subjects were randomly assigned to one of the two treatment sequences in a 1:1 ratio. Except for one subject who withdrew early from the study due to AEs (Increase of plasma alanine aminotransferase level) after single-dose administration in treatment period 1, the remaining 51 subjects finished the study and were all included in the pharmacokinetic and pharmacodynamic analysis.

**Table 1:** Baseline demographics and characteristics of study population.

Parameters	N	Mean	SD	Max	Min
Age (years)	52	30.1	8.10	50	20
Height (cm)	52	165.78	7.239	184.1	155.2
Weight (kg)	52	62.22	6.858	76.6	50.0
BMI (kg/m^2^)	52	22.62	1.777	26.0	19.6

*BMI* body mass index, *SD* standard deviation, *Max* maximum, *Min* minimum.

### Pharmacokinetics and bioequivalence

**Figure 1:**
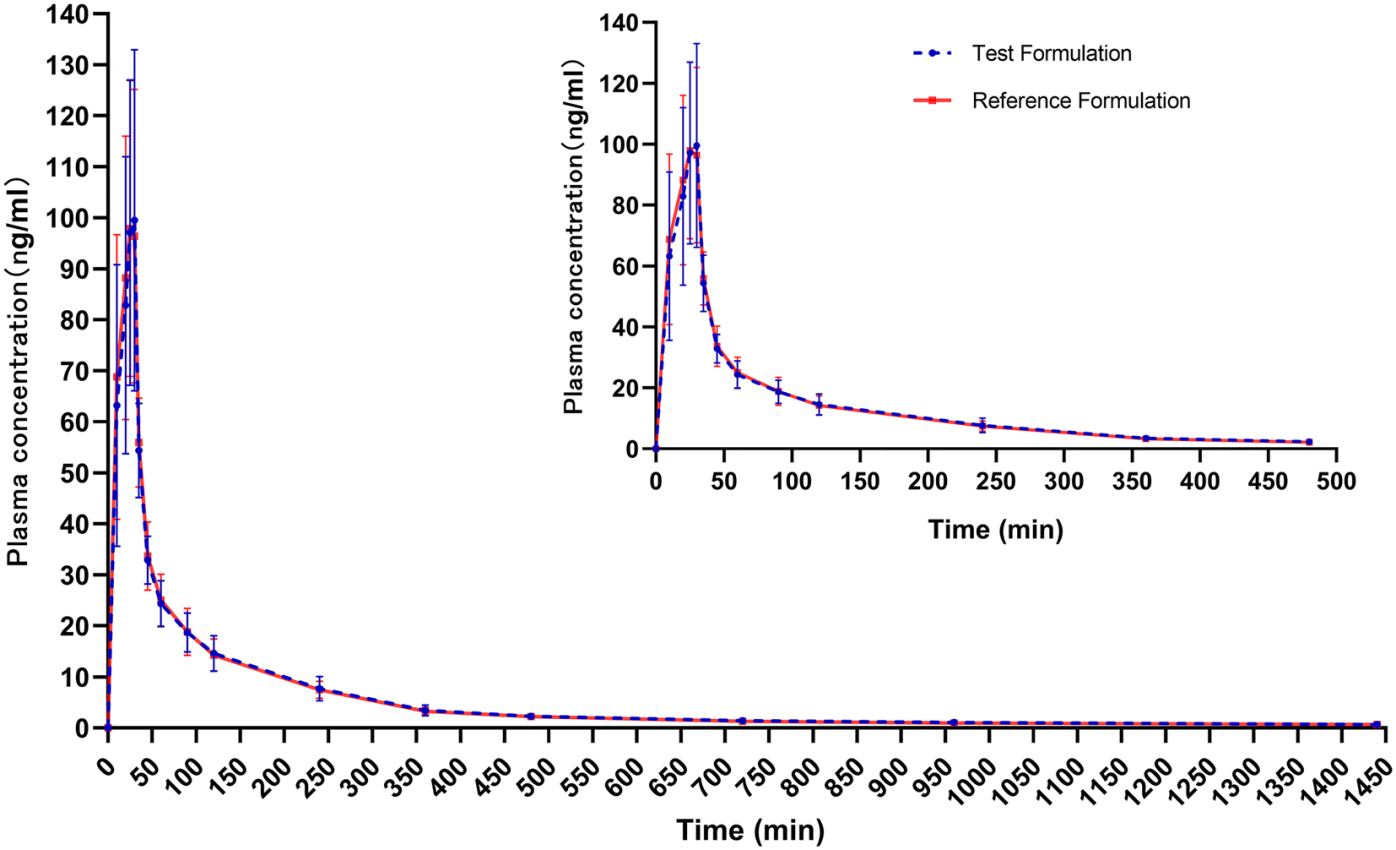
Mean plasma concentration–time curves for the test and reference formulations after a single dose of etomidate through the intravenous infusion pump in healthy subjects. n = 51. Bars represent SD.

Figure 1 shows the mean plasma concentration–time curves of the two formulations. The changing trends of the two plasma concentration–time curves is very similar. The primary pharmacokinetic parameters are listed in Table 2, which are expressed as mean ± SD. After administering the test formulation through the intravenous infusion pump, a C_max_ value of 106.25 ± 32.890 ng/mL of etomidate was rapidly achieved within 10.2–30.6 min; the mean AUC_0–t_, AUC_0–∞_ and t1/2 for etomidate were 7747.05 ± 1343.210 min ng/mL, 8282.72 ± 1369.054 min ng/mL, and 591.10 ± 222.861 min respectively. After administering the reference formulation through the intravenous infusion pump, a C_max_ value of 106.29 ± 26.508 ng/mL of etomidate was rapidly reached within 10.2–30.2 min; the mean AUC_0–t_, AUC_0–∞_ and t1/2 for etomidate were 7735.67 ± 1037.371 min ng/mL, 8333.36 ± 1169.744 min ng/mL, and 604.95 ± 231.461 min, respectively. These results indicated that there was no significant difference in the pharmacokinetic properties between the two formulations.

**Table 2:** Pharmacokinetic parameters of etomidate for the test and reference formulations after a single dose of etomidate through the intravenous infusion pump in healthy subjects (n = 51).

Pharmacokinetic parameters	Test	Reference
T_max_ (min)	30.03 (10.2, 30.6)	25.18 (10.2, 30.2)
C_max_ (ng/mL)	106.25 ± 32.890	106.29 ± 26.508
AUC_0–t_ (min ng/mL)	7747.05 ± 1343.210	7735.67 ± 1037.371
AUC_0–∞_ (min ng/mL)	8282.72 ± 1369.054	8333.36 ± 1169.744
t_1/2_ (min)	591.10 ± 222.861	604.95 ± 231.461

All values are expressed as mean ± SD except for T_max_ values, which are expressed as median (range).

*AUC*_*0–t*_ area under the plasma concentration–time curve from time zero to the time of the last measurable concentration, *AUC*_*0–∞*_ area under the plasma concentration–time curve from time zero to infinity, *C*_*max*_ maximum plasma drug concentration, *T*_*max*_ time to reach C_max_, *t*_*1/2*_ half-time of terminal elimination.

Table 3 shows the pharmacokinetic bioequivalence evaluation results of the two formulations. After ANOVA analysis, C_max_, AUC_0–t_, and AUC_0–∞_ showed no period, sequence, or formulation effect. The 90% CIs of the GLSM ratios of the test to the reference formulation, with C_max_ of 91.33–104.96%, AUC_0–t_ of 97.21–102.03%, and AUC_0–∞_ of 97.22–102.33%, were all within the accepted bioequivalence range of 80.00–125.00%. The coefficients of intra-subject variation (CV_w_%) for C_max_, AUC_0–t_, and AUC_0–∞_ were 21.17%, 7.31%, and 7.72%, respectively. The power of C_max_, AUC_0–t_, and AUC_0–∞_ was 99.9, > 99.9 and > 99.9, respectively The P value of the Non-parametric test for T_max_ between the two formulations was 0.0828 (> 0.05), with no significant statistical difference.

**Table 3:** Bioequivalence assessment of pharmacokinetic parameters of etomidate for the test and reference formulations after a single dose of etomidate through the intravenous infusion pump in healthy subjects (n = 51).

Parameters	Ration of GLSM (%)	90% CI	CV (%)	Power (%)
C_max_ (ng/mL)	97.91	91.33–104.96	21.17	99.9
AUC_0–t_ (min ng/mL)	99.59	97.21–102.03	7.31	> 99.9
AUC_0–∞_ (min ng/mL)	99.74	97.22–102.33	7.72	> 99.9

*AUC*_*0–t*_ area under the plasma concentration–time curve from time zero to the time of the last measurable concentration, *AUC*_*0–∞*_ area under the plasma concentration–time curve from time zero to infinity, *C*_*max*_ maximum plasma drug concentration, *CV* coefficients of variation, *GLSM* geometric least-squares mean.

### Pharmacodynamics and results

**Figure 2:**
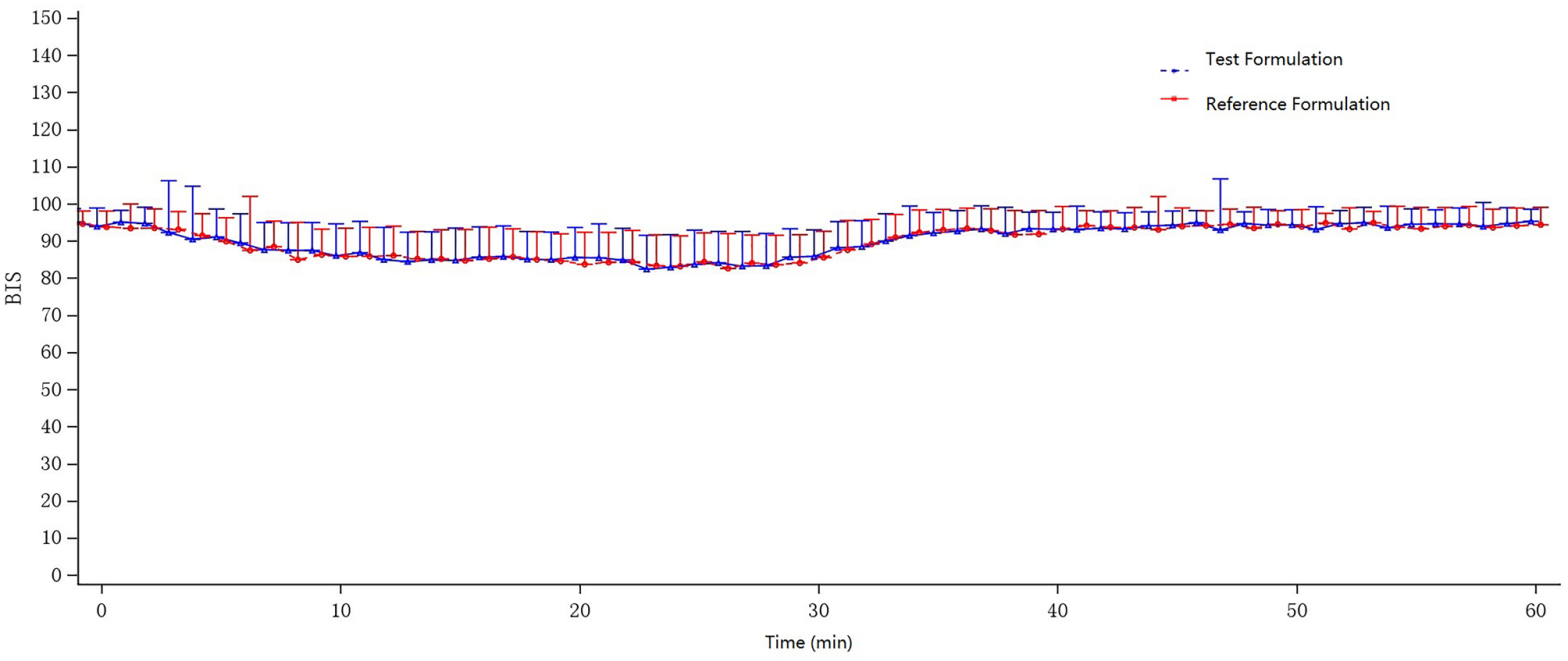
Mean BIS-time curves for the test and reference formulations after a single dose of etomidate through the intravenous infusion pump in healthy subjects. n = 51. Bars represent SD.

Figure 2 shows the mean BIS-time curves of the two formulations. There is not much difference in the trend of these two curves. Table 4 summarizes the results of the pharmacodynamic analysis. After administration of the test formulation through the intravenous infusion pump, t-BIS_min_ was 22.0 min (median), BIS_min_ was 72.2 ± 16.57 (CV% was 22.97%), BISAUC_0–60 min_ was 5396.94 ± 251.696 (CV% was 4.66%). After administration of the reference formulation through the intravenous infusion pump, t-BIS_min_ was 22.0 min (median), BIS_min_ was 69.5 ± 13.82 (CV% was 19.89%), BISAUC_0–60 min_ was 5383.30 ± 220.033 (CV% was 4.09%). These results indicated no significant difference in the pharmacodynamic properties between the two formulations.

**Table 4:** Pharmacodynamics parameters of etomidate for the test and reference formulations after a single dose of etomidate through the intravenous infusion pump in healthy subjects (n = 51).

Pharmacodynamics parameters	Test	Reference
t-BIS_min_ (min)	22.0 (3.0, 59.0)	22.0 (1.0, 58.0)
BIS_min_	72.2 ± 16.57	69.5 ± 13.82
BISAUC_0–60min_	5396.94 ± 251.696	5383.30 ± 220.033

All values are expressed as mean ± SD except for t-BIS_min_ values, which are expressed as median (range).

*BIS*_*min*_ the minimal BIS value, *BISAUC*_*0–60 min*_ the area under the BIS time curve from time 0 to 60 min after administration, *t-BIS*_*min*_ the time to the minimal BIS value.

Table 5 shows the pharmacodynamic statistical assessment results of the two formulations. The 90% CIs of the GLSM ratios of the test to the reference formulation, with BIS_min_ of 102.28–110.69% and BISAUC_0–60 min_ of 99.23–101.17%, both within the accepted common criteria for bioequivalence ranged from 80.00 to 125.00%. The CV_w_% for BIS_min_ and BISAUC_0–60 min_ were 11.72%, and 2.92%, respectively. The power of BIS_min_ and BISAUC_0–60 min_ were > 99.9. The P value of the Non-parametric test for t-BIS_min_ between the two formulations was 0.3320 (> 0.05), with no significant statistical difference.

**Table 5:** Statistical bioequivalence assessment of pharmacodynamic parameters of etomidate for the test and reference formulations after a single dose of etomidate through the intravenous infusion pump in healthy subjects (n = 51).

Parameters	Ration of GLSM (%)	90% CI	CV (%)	Power (%)
BIS_min_	106.40	102.28–110.69	11.72	> 99.9
BISAUC_0–60min_	100.19	99.23–101.17	2.92	> 99.9

*BIS*_*min*_ the minimal BIS value, *BISAUC*_*0–60 min*_ the area under the BIS time curve from time 0 to 60 min after administration, *CV* coefficients of variation, *GLSM* geometric least-squares mean.

Meanwhile, the sedation depth of the subjects was evaluated by anesthesiologists with modified MOAA/S score in the study. The evaluation was conducted within 5 min before the beginning of administration, and every 15 min within 60 min after administration until the end of sedation. That is to say, the subjects were given five modified MOAA/S scores in every period during the whole trial. Table 6 summarizes the results of the descriptive summary analysis for the Modified MOAA/S score. After a single dose of the test formulation, 7 subjects were grade 2 in the second evaluation (13.7%, 7/51) and5 subjects were grade 2 in the third evaluation (9.8%, 5/51), respectively, all other evaluations were grade 1. After a single dose of the reference formulation, 5 subjects were grade 2 (9.8%, 5/52) and 1 subject was grade 3 (2.0%, 1/52) in the second evaluation, and 10 subjects were grade 2 (19.6%, 10/52) in the third evaluation, respectively, all other evaluations were grade 1. These results indicated that etomidate’s sedative and hypnotic effects also occurred in some healthy subjects after low dose administration, and the difference in the sedative and hypnotic effects was minimal between the two formulations.

**Table 6:** Modified MOAA/S scores of etomidate for the test and reference formulations after a single dose of etomidate through the intravenous infusion pump in healthy subjects (n = 51).

Scoring times	Grading	Result	
		Test	Reference
1	1, n (%)	51 (100%)	51 (100%)
	2, n (%)	0	0
	3, n (%)	0	0
	4, n (%)	0	0
	5, n (%)	0	0
2	1, n (%)	44 (86.3%)	45 (88.2%)
	2, n (%)	7 (13.7%)	5 (9.8%)
	3, n (%)	0	1 (2.0%)
	4, n (%)	0	0
	5, n (%)	0	0
3	1, n (%)	46 (90.2%)	41 (80.4%)
	2, n (%)	5 (9.8%)	10 (19.6%)
	3, n (%)	0	0
	4, n (%)	0	0
	5, n (%)	0	0
4	1, n (%)	51 (100%)	51 (100%)
	2, n (%)	0	0
	3, n (%)	0	0
	4, n (%)	0	0
	5, n (%)	0	0
5	1, n (%)	51 (100%)	51 (100%)
	2, n (%)	0	0
	3, n (%)	0	0
	4, n (%)	0	0
	5, n (%)	0	0

Scoring criteria of modified MOAA/S scores:

1. Grade 1: alert, responds readily to name spoken in normal tone;

2. Grade 2: lethargic response to name spoken in normal tone;

3. Grade 3: no response to normal calls, but responds only after name is called loudly and/or repeatedly;

4. Grade 4: no response to loud and/or repeated calls, but responds only after mild prodding or shaking;

5. Grade 5: no response to mild prodding or shaking, but responds to noxious stimulation.

### Safety profile

The safety analysis set included all 52 subjects who participated in the study and assessed the safety of etomidate. Table 7 summarizes the AEs of the two formulations. During the study period, 13 subjects reported 20 cases of AE, with an incidence of 12.6% (13/103). The incidence of AEs of the test produce was 9.8% (5/51), while that of the reference produce was 15.4% (8/52). Except for 1 case AE was moderate in intensity, all other AEs were mild in intensity, and all subjects recovered spontaneously without treatment. Throughout the entire study process, none of the subjects experienced serious adverse events. These safety results suggested that both formulations of etomidate had good safety and tolerability in healthy subjects.

**Table 7:** AEs in healthy subjects in the study.

	Test (N = 51)		Reference (N = 52)		Total (N = 103)	
	AE count	N (%)	AE count	N (%)	AE count	N (%)
Total	9	5 (9.8)	11	8(15.4)	20	13 (12.6)
Metabolic and nutritional diseases	2	1 (2.0)	2	2 (3.8)	4	3 (2.9)
Hypertriglyceridemia	1	1 (2.0)	1	1 (1.9)	2	2 (1.9)
Hyperuricemia	1	1 (2.0)	1	1 (1.9)	2	2 (1.9)
Results of inspection	6	4 (7.8)	5	4 (7.7)	11	8 (7.8)
Leukocyte count decreased^c^	1	1 (2.0)	–	–	1	1 (1.0)
Leukocyte count increased	1	1 (2.0)	1	1 (1.9)	2	2 (1.9)
Lmphocyte count decreased	1	1 (2.0)	–	–	1	1 (1.0)
Neutrophils count decreased	1	1 (2.0)	–	–	1	1 (1.0)
Neutrophils count increased	1	1 (2.0)	1	1 (1.9)	2	2 (1.9)
ALT increased	–	–	1	1 (1.9)	1	1 (1.0)
Blood creatinine increased	–	–	1	1 (1.9)	1	1 (1.0)
Urinary white blood cell positivity^a^	1	1 (2.0)	–	–	1	1 (1.0)
Urinary protein positive	–	–	1	1 (1.9)	1	1 (1.0)
Various neurological diseases	1	1 (2.0)	2	1 (1.9)	3	2 (1.9)
Pre syncope stage	1	1 (2.0)	1	1 (1.9)	2	2 (1.9)
Dizziness	–	–	1	1 (1.9)	1	1 (1.0)
Psychiatric category	–	–	1	1 (1.9)	1	1 (1.0)
Injecting fear^b^	–	–	1	1 (1.9)	1	1 (1.0)
Gastrointestinal diseases	–	–	1	1 (1.9)	1	1 (1.0)
Nausea	–	–	1	1 (1.9)	1	1 (1.0)

Data are presented as AE count and Number of subjects in respective categories of AE (%), respectively. Percentages are based on the number of subjects allocated to treatment.

*AE* adverse event, *N* number of subjects, *ALT* alanine aminotransferase.

^a^AE of urinary white blood cell positivity.

^b^AE of injecting fear were recorded was not related to the study drug, all other AEs were reported as adverse drug reaction.

^c^AE of leukocyte count decreased was moderate in intensity, all other AEs were mild in intensity.

## Discussion

Although etomidate is widely used for induction of general anesthesia for adults, infants and young children over 6 months old, there is no published information, guiding principles and related articles on the pharmacokinetics and bioequivalence of etomidate free-fat emulsion injection in healthy subjects. The present study fully compared and evaluated the pharmacokinetic and pharmacodynamic properties and bioequivalence of two formulations in healthy Chinese subjects, and it is also the first report on the bioequivalence study of etomidate medium/long-chain fat emulsion in healthy subjects. Up to now, the United States Food and Drug Administration (FDA) and European Medicines Agency (EMA) have not yet issued guidance on etomidate. Moreover, there are not much literature available for reference. Therefore, when conducting clinical research on etomidate bioequivalence study, how to scientifically and reasonably design the dosage, administration method, pharmacokinetic blood collection points, and the number of subjects is a huge challenge for the clinical researchers.

When designing the dosage of etomidate, we referred to FDA Draft Guidance on Propofol^7^, which is a drug similar to etomidate. It is mentioned in FDA guideline that propofol should be administered as a slow intravenous infusion at a rate of 30 μg/kg/min with monitoring and any necessary intervention by an anesthesiologist or nurse anesthetist throughout the infusion; each subject should receive an infusion for 30 min. In addition, according to the relevant literature^8^, 0.5 mg/kg etomidate and 2.5 mg/kg propofol can reach the same anesthesia level. Therefore, in the study, etomidate was designed to be administered as a slow intravenous infusion at a rate of 5 μg/kg/min for 30 min.

In the design of pharmacokinetic blood collection points, based on the limited known pharmacokinetic characteristics of etomidate, we conducted two pilot trials in 2 and 8 healthy subjects respectively, before the study in 52 healthy subjects. C_max_ and T_max_ of etomidate medium/long chain fat emulsion injection are mainly determined by formulation factors, and there is no accurate data about etomidate medium/long chain fat emulsion injection. According to the general formulation characteristics of fat emulsion injection, T_max_ usually appears immediately or within 30 min after the end of infusion. The single bolus pharmacokinetic profile of plasma etomidate concentration is described by a three compartment model^9^, the initial distribution half-life of etomidate was 2.7 min, the second distribution half-life was 29 min, and the elimination half-life was 2.9–5.3 h. Based on the results of pilot trial 1 and pilot trial 2, the pharmacokinetic blood samples in the study were finally designed to collect at 0 h (within 1 h before the dose) and 10, 20, 25, 30, 35, and 45 min, and 1.0, 1.5, 2.0, 4.0, 6.0, 8.0, 12.0, 16.0, 24.0 h after drug administration. The study results showed further that the design of pharmacokinetic blood collection points of the study is reasonable, which can cover the distribution, metabolism, and excretion process of etomidate in vivo, and can well reflect the pharmacokinetic characteristics of etomidate in healthy subjects. In addition, we compared the results of T_max_ and t_1/2_ in the study with previous reports. T_max_ of the two etomidate formulations was about 30 min, which were consistent with the general characteristics of fat emulsion injection, that is, T_max_ usually appears immediately or within 30 min after the end of infusion. t_1/2_ of the two etomidate formulations was about 600 min, which is longer than the 2–5 h reported in previous literature^1^. The reason for this difference in t_1/2_ may be related to the more sensitive blood drug concentration detection methods currently used, which can detect lower blood drug concentrations in the elimination phase.

When estimating the sample size of subjects, the coefficient of variation of etomidate is a key data point, but we had no any useful literature to refer to. Therefore, we had to consider it comprehensively based on the relative regulations and the results of the pilot trial 2 conducted on 8 healthy subjects. We set the Intra-individual coefficient of variation (Intra-CV) of C_max_ for etomidate at 32.8% according to the result of pilot tiral 2, the significance level at 0.05, and the power value at 0.8, respectively. In addition, we set the geometric mean ratio of the two formulations to 0.953 and the bioequivalence range as 80.00–125.00%. As a result, after calculation, the estimated number of subjects required for the study was 45. In addition, considering the possibility of early withdrawal of individual subjects and supposing a drop-off rate of 15%, we finally enrolled 52 subjects in the current study. As shown in Table 3, the power of C_max_, AUC_0–t_, and AUC_0–∞_ was approximately 100%, indicating that our expected sample size was correct and reasonable. The CVw% for C_max_, AUC_0–t_, and AUC_0–∞_ in the study were 21.17%, 7.31%, and 7.72%, respectively, suggesting that etomidate has no high variability from the perspective of pharmacokinetics. The intra-CV of C_max_ (21.17%) in the study was lower than the intra-CV of C_max_ (32.8%) in pilot tiral 2. The reason for this difference may be related to the smaller number of subjects in the pilot tiral 2, which was only 8 subjects. The small difference in intra-CV was reasonable and acceptable.

As mentioned, there is no formal regulatory guidance for the study of etomidate BE (PK or PD), so we need to carefully consider it when conducting bioequivalence evaluation. Although the FDA and EMA have not yet released individual drug BE guidelines for etomidate, the National Medical Products Administration (NMPA) of China released a draft of technical guidelines for the bioequivalence study of etomidate medium/long-chain fat emulsion injection in July 2023^10^, which can provide useful reference for BE evaluation of etomidate. The guiding principle mentions that the C_max_, AUC_0–t_, and AUC_0–∞_ of etomidate are used as bioequivalence evaluation indicators. The average bioequivalence (ABE) method is used for evaluation, and the bioequivalence acceptance standard is the GLSM of C_max_, AUC_0–t_, and AUC_0–∞_ of the test and reference formulations, with a 90% CI between 80.00 and 125.00%. Therefore, according to this evaluation standard, the 90% CI of the GLSM ratios of C_max_, AUC_0–t_, and AUC_0–∞_ of the test and reference formulations in this study are within the range of 80.00–125.00%, which meet the requirements of pharmacokinetic bioequivalence. The P value of T_max_ between the two formulations had no significant statistical difference, further supporting the conclusion of pharmacokinetic equivalence in vivo between the two formulations. Meanwhile, we noticed that the FDA's draft guidance on propofol required additional "characterization studies" including global size distribution, zeta potential profile, and isoelectric point et al., in addition to an in vivo pharmacokinetic bioequivalence study, when determining the bioequivalence of propofol. But this requirement for propofol was established in 2016, and it is currently unknown whether it also applies to BE studies of etomidate. In addition, the draft of technical guidelines for the bioequivalence study of etomidate released by NMPA of China in July 2023 did not include this requirement for these additional "characterization studies". Therefore, when evaluating etomidate BE, whether it is necessary to conduct additional "characterization studies" remains to be confirmed by the drug regulatory authorities.

Furthermore, while conducting the pharmacokinetic bioequivalence study, we also conducted exploratory study on pharmacodynamic evaluation of etomidate. In the pharmacodynamic study of etomidate, we selected BIS as the main pharmacodynamics index and used BIS values to calculate the main pharmacodynamics parameters. BIS is a number obtained by processing the power and frequency of electroencephalogram (EEG) using fast Fourier transform (FFT) and dual-frequency technology. The BIS value is represented by 0–100. The higher the BIS value, the more conscious the patient will be. BIS value can reflect the functional status of cerebral cortex, which has been considered as a sensitive and accurate objective index to evaluate the state of consciousness of patients. When the BIS value is 100, it represents the awake state. With the deepening of sedation level, BIS value gradually decreases. BIS values of about 70 represents mild sedation, about 60 for BIS represents moderate sedation, 40–50 for BIS represents deep sedation, and if BIS value drops to 0, it represents EEG suppression. In the study, mean BIS_min_ values obtained from BIS monitoring system of 51 healthy subjects were between 60 and 70. That is to say, after administration of etomidate, some subjects entered a mild sedation state, while others subjects entered a moderate sedation state. Although the dosage of etomidate in the study was much lower than the clinical anesthetic dosage, sedation still occurred in healthy subjects. Some published studies on propofol BE, although designed with pharmacodynamic parameters, have not specified equivalent acceptance criteria. Therefore, we referred to the evaluation criteria of conventional pharmacokinetic BE and pharmacodynamic BE and selected 80.00–125.00% as pharmacodynamic equivalent interval for etomidate. When the 90% CIs of the GLSM ratios of the pharmacodynamic parameters of the test and reference formulations fell within the equivalent interval of 80.00–125.00%, we considered that the pharmacodynamic parameters of the two formulations meet the common criteria of bioequivalence. The results of the pharmacodynamic analysis in this study indicated that using BIS as a pharmacodynamics index to evaluate the pharmacodynamics BE has certain feasibility, which may provide some useful reference for bioequivalence of etomidate, and the pharmacodynamic results also can serve as a basis for further supporting clinical efficacy.

**Figure 3:**
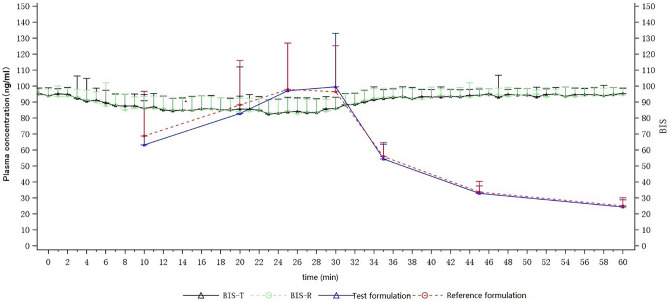
Mean Concentration and BIS-time curves for the test and reference formulations after a single dose of etomidate through the intravenous infusion pump in healthy subjects. n = 51. Bars represent SD.

We also analyzed the pharmacokinetics and pharmacodynamics results of etomidate. As shown in Fig. 3, there was a certain correlation between blood drug concentration and BIS value after the etomidate administration. With the increase of drug concentration, BIS value had a downward trend. Meanwhile, the time to the minimal BIS value (t-BIS_min_) was close to the time to maximum plasma concentration (T_max_). However, the dose–response relationship of etomidate PK-PD could not be accurately calculated due to the small dosage of etomidate. Further investigations are needed to evaluate the dose–response relationship of etomidate PK-PD.

Except for one AE with moderate severity, all other AEs were mild. All AEs were quickly resolved without treatment. No significant difference between the two formulations was found in the incidence and severity of AEs. Therefore, the two formulations have good safety and tolerability in healthy subjects.

## Conclusion

The current study demonstrated that the etomidate test and reference formulations had similar pharmacokinetic and pharmacodynamic characteristics in vivo after intravenous infusion in healthy Chinese subjects, which the main PK and PD parameter ratios of the test to the reference formulation all fall in the common BE criteria of 80.00–125.00%. Meanwhile, the two formulations showed good tolerance and did not present any severe medication safety issues. Moreover, the current research can provide important references for the bioequivalence study of etomidate in terms of research design, sample size, and blood collection points.

## Data Availability

The datasets used and analyzed during the current study available from the corresponding author on reasonable request.
